# Silencing of B7-H3 increases gemcitabine sensitivity by promoting apoptosis in pancreatic carcinoma

**DOI:** 10.3892/ol.2013.1118

**Published:** 2013-01-08

**Authors:** XIN ZHAO, GUANG-BO ZHANG, WEN-JUAN GAN, FENG XIONG, ZHI LI, HUA ZHAO, DONG-MING ZHU, BIN ZHANG, XUE-GUANG ZHANG, DE-CHUN LI

**Affiliations:** 1 Departments of General Surgery, The First Affiliated Hospital of Soochow University, Suzhou, Jiangsu 215006, P.R. China; 2 Clinic Immunology, The First Affiliated Hospital of Soochow University, Suzhou, Jiangsu 215006, P.R. China; 3 Pathology, The First Affiliated Hospital of Soochow University, Suzhou, Jiangsu 215006, P.R. China; 4 Oncology, The First Affiliated Hospital of Soochow University, Suzhou, Jiangsu 215006, P.R. China; 5 Intervention Radiology, The First Affiliated Hospital of Soochow University, Suzhou, Jiangsu 215006, P.R. China; 6 Nuclear Medicine, The First Affiliated Hospital of Soochow University, Suzhou, Jiangsu 215006, P.R. China

**Keywords:** pancreatic carcinoma, B7-H3, chemoresistance, shRNA

## Abstract

In numerous types of cancer, the expression of a novel member of the B7 ligand family, the B7-H3 immunoregulatory protein, has been correlated with a poor prognosis. In the present study, we investigated the role of B7-H3 in chemoresistance in pancreatic carcinoma. Silencing of B7-H3, through lentivirus-mediated delivery of stable short hairpin RNA, was observed to increase the sensitivity of the human pancreatic carcinoma cell line Patu8988 to gemcitabine as a result of enhanced drug-induced apoptosis. Overexpression of B7-H3 caused the cancer cells to be more resistant to the drug. Subsequently, we investigated the underlying mechanisms of B7-H3-mediated gemcitabine resistance, and found that the levels of survivin decreased in cells in which B7-H3 had been knocked down. *In vivo* animal experiments demonstrated that tumors in which B7-H3 had been knocked down displayed a slower growth rate compared with the control xenografts. Notably, gemcitabine treatment led to a strong antitumor activity in mice with tumors in which B7-H3 had been knocked down; however, this effect was only marginal in the control group. Furthermore, survivin expression was weak in gemcitabine-treated tumors in which B7-H3 had been knocked down and apoptosis was increased, as revealed by terminal deoxynucleotidyltransferase-mediated dUTP-biotin nick-end labeling (TUNEL) staining. In summary, the present study demonstrated that B7-H3 induces gemcitabine resistance in pancreatic carcinoma cells, at least partially by downregulating survivin expression. These results provide novel insights into the function of B7-H3 and encourage the design and investigation of approaches targeting this protein in treating pancreatic carcinoma.

## Introduction

Pancreatic carcinoma is one of the most aggressive human malignant tumors and a leading cause of cancer-related mortality worldwide (1). Its anatomical complexity and late diagnosis have led to a low resectability rate of 10–20%. Due to the poor 5-year survival rate, the incidence and mortality of the disease are approximately equivalent (2). Although recent significant advances in cancer therapy, including the introduction of novel chemotherapeutic agents, have significantly impacted the overall survival time of pancreatic cancer patients, complete recovery is extremely rare (3). Therefore, it is essential that therapeutic regimens be developed to inhibit tumor growth and increase the chemosensitivity of chemotherapeutics; new approaches, including gene therapy, are required to improve treatment results (4,5).

B7-H3 is a novel member of the B7 family (6). B7-H3 is not expressed in quiescent lymphocytes and may be induced in activated dendritic cells, monocytes and T cells (7–9). The protein is overexpressed in several human cancer types, including breast, renal cell, urothelial cell, prostate, lung and pancreatic cancer. Previous studies have demonstrated a correlation between high expression of B7-H3 and a poor outcome in patients with these types of cancer (10–15). However, the precise role of B7-H3 in tumors remains unclear. As metastasis and proliferation are closely correlated with chemoresistance, we aimed to investigate the role of B7-H3 in the sensitivity of pancreatic carcinoma cells to gemcitabine, and the possible underlying mechanisms.

## Materials and methods

### Reagents

Antiobdies, including anti-human B7-H3, anti-survivin and an antibody against GAPDH, were purchased from R&D Systems (Minneapolis, MN, USA). The horseradish peroxidase-conjugated secondary anti-mouse, anti-rabbit and anti-goat antibodies were purchased from Bio-Rad (Hercules, CA, USA). Gemcitabine was purchased from Lilly France, Inc. (Neuilly, France), while TRIzol reagent and Moloney murine leukemia virus (MMLV) were purchased from Gibco-BRL (Carlsbad, CA, USA). *Taq* DNA polymerase, dNTPs and DNA markers were obtained from Takara Bio, Inc. (Shiga, Japan).

### Clinical specimens from patients

This study was approved by the Ethics Committee of The First Affiliated Hospital of Soochow University for Clinical Investigation. Forty patients with pancreatic carcinoma who underwent radical resection surgery were included in the study. Patients were excluded from analysis if they had received chemotherapy or radiation therapy prior to surgery, or if they had undergone previous pancreatic surgery. Pancreatic carcinoma specimens were obtained during surgery, following written consent. Normal pancreatic tissue specimens (confirmed histopathologically and distant to the tumor) were simultaneously obtained as controls. Following dissection under sterile conditions, each tissue sample was fixed in 10% buffered methanal for immunohistochemical estimation of B7-H3 expression.

### Cells and cell culture

The pancreatic carcinoma cell line Panc-1 was purchased from the Chinese Academy of Science Cell Bank, and the Patu8988 cell line was provided by Professor Chang-Geng Ruan from the Jiangsu Provincial Institute of Hematology, China. Panc-1 cells were cultured in Dulbecco’s modified Eagle’s medium (Sigma, St. Louis, MO, USA) and Patu8988 cells were cultured in RPMI-1640 medium (Gibco-BRL), and all media were supplemented with 10% fetal bovine serum (Atlanta Biologicals, Inc., Lawrenceville, GA, USA) and 1% penicillin-streptomycin (Gibco-BRL) at 37°C and 5% CO_2_.

### Generation of stable cell lines

Small hairpin RNA (shRNA) of the human B7-H3 (GenBank, NM_001024736) lentivirus gene transfer vector encoding the green fluorescent protein (GFP) sequence was constructed by Shanghai Genechem Co. (Shanghai, China). The targeting sequence of shB7-H3 was: 5′-GAGCAGGGCTTGTTTGATGTG-3′, and it was confirmed by sequencing. The recombinant lentivirus of small interfering RNA targeting B7-H3 (LV-shB7-H3) and the non-targeted control mock lentivirus (LV-NC) were prepared and titered to 5×10^9^ TU/ml (transfection unit). Cells were subcultured at 5×10^4^ cells/well in 6-well tissue culture plates overnight. The viral supernatant was then added to cells at a multiplicity of infection (MOI) of 10 with Enhanced Infection Solution (Shanghai Genechem Co., Shanghai, China) and 5 *μ*g/ml polybrene. Real-time reverse transcription-polymerase chain reaction (RT-PCR) and western blot analysis were performed to confirm the knockdown of B7-H3 mRNA and protein, respectively, in those transfectants.

### Real time RT-PCR

RT-PCR was performed to confirm the knockdown of B7-H3 mRNA in the transfectants. Total RNA was collected using TRIzol reagent, according to the manufacturer’s instructions. The concentration and purity of the total RNA were detected with an ultraviolet spectrophotometer and the mRNA was reversely transcribed into cDNA with MMLV. Quantitative real-time PCR assays were conducted using SYBR Green realtime PCR Master Mix and real-time PCR amplification equipment. GAPDH was used as an internal control. The PCR conditions consisted of one cycle at 95°C for 15 sec, followed by 45 cycles at 95°C for 5 sec and at 60°C for 30 sec. The primer sequences were as follows: Sense, 5′-CTCTGCCTTCTCACCTCTTTG-3′ and antisense, 5′-CCTTGAGGGAGGAACTTTATC-3′ for B7-H3 (134 bp); sense, 5′-TGACTTCAACAGCGACACCCA-3′ and antisense, 5′-CACCCTGTTGCTGTAGCCAAA-3′ for GAPDH (121 bp).

### Flow cytometry

The infected positive clones were isolated by sorting flow cytometry (FCM) according to GFP expression. Furthermore, stable clones of each GFP expression rate group were detected by FCM. The infected cells comprised the LV-shB7-H3 and LV-NC groups, and the non-infected Patu8988 cells were the control group. These three groups were used in the following experiments.

### In vitro growth inhibition

Cells (1×10^4^ cells/well) were initially plated in triplicate in 96-well culture plates. After 24 h, the medium was replaced with fresh medium with or without gemcitabine and cells were incubated for 72 h. Cell viability following treatment with various concentrations of gemcitabine was assessed by 3-(4,5-dimethylthiazol-2-yl)-5-(3-carboxymethoxyphenyl)-2-(4-sulfophenyl)-2H-tetrazolium (MTS) assay. Cell viability was determined using the Cell Titer 96 Aqueous One Solution Cell Proliferation Assay kit (Promega, Madison, WI, USA).

### Annexin V staining

Cells (3×10^5^ cells/dish) were grown in triplicate in 60-mm dishes with exposure to 5.00 *μ*mol/l gemcitabine for 0, 48 and 72 h. Cells were then collected and processed as described in the Annexin V-FITC Apoptosis Detection kit I manual (Invitrogen Life Technologies; Carlsbad, CA, USA). However, we discovered that the GFP expression was capable of interfering with the FITC analysis assessed by flow cytometry, as both express a similar green fluorescence. Therefore, propidium iodide (PI) reagent was used only to detect apoptosis, and the FITC reagent was not used.

### Terminal deoxynucleotidyltransferase-mediated dUTP-biotin nick-end labeling (TUNEL) assay for cells

A TUNEL assay was performed using recombinant terminal transferase (TdT) and biotin-16-dUTP (Roche Diagnostics GmbH, Mannheim, Germany). Cells were treated with 5.00 *μ*mol/l gemcitabine for 0, 48 and 72 h. Cells were processed according to the manufacturer’s instructions and were analyzed by flow cytometery (BD Biosciences, Franklin Lakes, NJ, USA). Each experiment was repeated three times.

### Western blot analysis

Cells were washed twice and lysed on ice. Following centrifugation, the supernatants were collected. Protein concentrations were determined by the Bio-Rad Dc Protein Assay system. Samples were then separated by 10% SDS-PAGE and transferred onto a polyvinylidene fluoride (PVDF) membrane. Membranes were blocked and incubated with primary antibodies, such as anti-B7-H3 (1:50 dilution), anti-survivin (1:50 dilution) or anti-GAPDH (1:100 dilution) antibodies, at 4°C overnight. After three washes, the membranes underwent hybridization with a goat-anti-mouse IgG conjugated with horseradish peroxidase (1:5,000 dilution) for 2 h at room temperature. Following further washing, reactive bands were visualized using ECLTM Western Blot Detection Reagents with exposure to X-ray film for 30–120 sec. The intensities of the bands were calculated by densitometric analysis using Image J software.

### In vivo studies

Six groups of six male Balb/c nude mice, 5–6 weeks old and 20 g in weight, were bred in aseptic specific pathogen-free (SPF) conditions and maintained at a constant humidity and temperature (25–28°C). Animal experiments were conducted according to protocols approved by the Animal Care and Use Committee of Soochow University and were in compliance with the guidelines regarding animal welfare of the China National Committee for Animal Experiments. Cells (2×10^7^; LV-shB7-H3, LV-NC or Patu8988 cells) in 0.2 ml normal sodium were injected subcutaneously into the right inguinal region of nude mice. For therapy experiments, gemcitabine was dissolved in normal sodium (0.02 mmol/ml) and a single dose of 2.5 ml/kg of the solution was injected intravenously into the tail vein when the mean tumor diameter was 5–6 mm (day 0). The untreated mice groups received only the solvent. The tumor size was measured twice a week with calipers, and the volume was determined using the simplified formula of a rotational ellipsoid (L×W^2^×0.5). Growth curves were constructed, and the data are presented as mean ± standard deviation. Tumors were harvested from mice seven weeks after treatment. B7-H3 and survivin expression were detected by immunohistochemistry, and a TUNEL assay was performed in the tumor xenografts.

### Immunohistochemistry

Clinical specimens and the tumor xenografts were used for immunohistochemical studies. Specimens were fixed in formalin overnight and embedded in paraffin. Series of 4-*μ*m sections were prepared for immunohistological staining. Tissue sections were quenched for endogenous peroxidase with freshly prepared 3% H_2_O_2_ with 0.1% sodium azide and then placed in an antigen retrieval solution for 15 min. Following incubation in the casein block, primary antibodies, including anti-B7-H3 (1:50 dilution) or anti-survivin (1:50 dilution), were applied to the sections for 1 h at room temperature. This was followed by incubation with the second antibody and extravidin-conjugated horseradish peroxidase. The immune reaction was counterstained with hematoxylin, then dehydrated and mounted. Sections were subsequently evaluated for the presence of brown diaminobenzidine precipitates indicative of positive reactivity by microscopy. The brown staining around the nucleus was interpreted as positive reactivity for B7-H3 and survivin.

### TUNEL assay for tumor xenografts

Apoptotic tumor cells were detected *in vivo* with the TUNEL method, using an *In Situ* Cell Death Detection kit (Roche Diagnostics GmbH). The assay was performed according to the manufacturer’s instructions. Briefly, following routine deparaffinization and treatment with H_2_O_2_ (3%), sections were digested with proteinase K (20 *μ*g/ml; pH, 7.4; 12 min) at 25°C and incubated with the reaction mixture (1:40; 60 min) at 37°C. Incorporated fluorescein was detected with horseradish peroxidase following a 30 min incubation at 37°C, and subsequently dyed with 3,3′-diaminobenzidine (DAB). A brown nucleus was considered to indicate a positive apoptotic cell.

### Statistical analysis

Pancreatic carcinoma and normal pancreas tissue B7-H3 expression in the immunohistochemical staining was compared and assessed using the χ^2^ test. The remainder of the data are presented as mean ± standard deviation. Statistical comparisons were performed using a Student’s t-test. All P-values were determined by two-sided tests, and P<0.05 was considered to indicate a statistically significant difference. Analyses were performed using SPSS 13.0 software (SPSS, Inc., Chicago, IL, USA).

## Results

### B7-H3 expression in pancreatic carcinoma cell lines and clinical specimens

**Figure 1:**
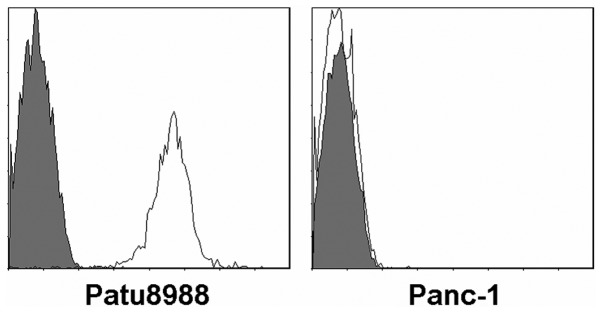
B7-H3 expression in pancreatic carcinoma cell lines. Expression of B7-H3 was analyzed by flow cytometry (FCM) in two human pancreatic carcinoma cell lines. As indicated, B7-H3 is present in Patu8988 cells, but not in Panc-1 cells.

**Figure 2:**
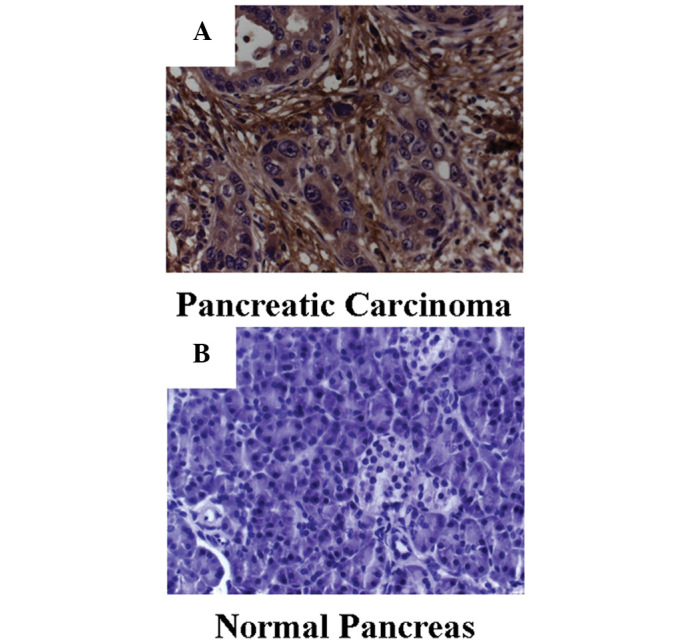
Immunohistochemical staining for B7-H3 in pancreatic carcinoma clinical specimens. (A) Overexpression in the tumor tissue. (B) Low expression in the normal pancreatic tissue. Magnification, ×400.

The expression of B7-H3 in human pancreatic carcinoma tissues from primary tumors has been demonstrated (15,16). In the present study, we investigated two pancreatic carcinoma cell lines, Panc-1 and Patu8988 (Fig. 1). As assessed by FCM, the B7-H3 protein was present in Patu8988 cell lines but not in Panc-1 cell lines. Samples from pancreatic cancer patients were also included, and immunohistochemical staining revealed that B7-H3 was significantly overexpressed in the tumor tissue (χ^2^, 57.313; P<0.001). B7-H3 expression was detected in >50% of cells in 31 tumor specimens, but not in the normal pancreas tissue specimens. In six of the tumor specimens and five of the normal pancreas tissue specimens, 25–50% of cells stained positively. In three of the tumor specimens and 35 of the normal pancreas tissue specimens, <25% positive staining was observed (Fig. 2).

### Silencing of B7-H3 enhances gemcitabine-induced cytotoxicity in pancreatic carcinoma

**Figure 3:**
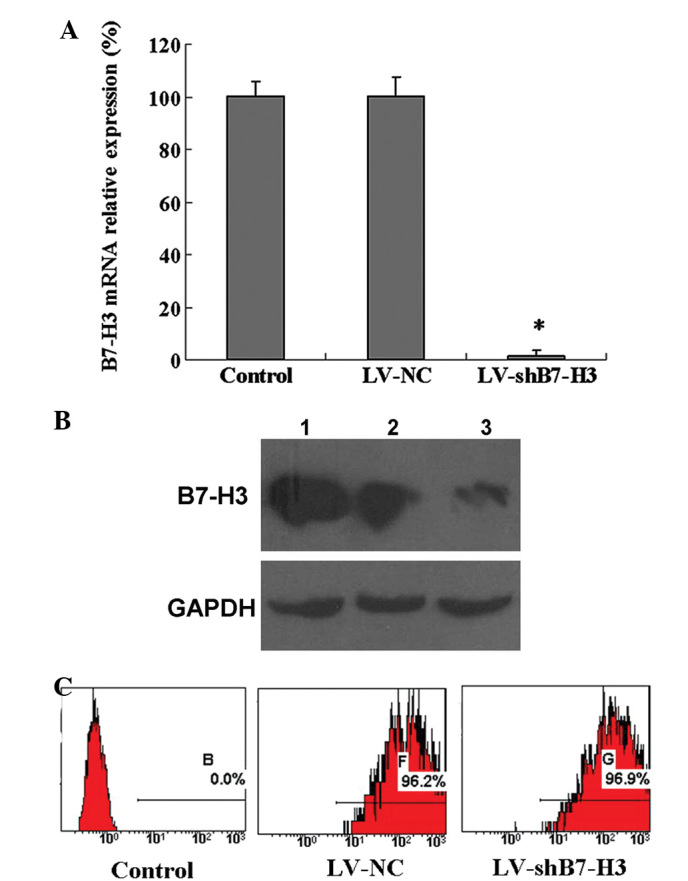
Silencing effect of B7-H3. (A) Knockdown of B7-H3 mRNA demonstrated by real-time RT-PCR. Relative expression of B7-H3 mRNA was analyzed using the 2^−ΔΔCt^ method. B7-H3 mRNA expression was significantly inhibited in the Patu8988 cell lines in which B7-H3 had been knocked down (the LV-shB7-H3 group) (^*^P<0.01 vs the control group). (B) Knockdown of B7-H3 protein demonstrated by western blot analysis. B7-H3 protein expression levels were markedly downregulated compared with the other two groups [1, control; 2, the non-targeted control mock lentivirus-infected Patu8988 cells (LV-NC); 3, LV-shB7-H3]. (C) Following sorting, GFP expression was assessed by flow cytometry. Infected positive cell ratios of LV-NC and LV-shB7-H3 were 96.2 and 96.9%, respectively.

To study the possible involvement of B7-H3 in the sensitivity of pancreatic carcinoma cells to gemcitabine, shRNA was used to create a stable B7-H3 knockdown cell variant derived from the Patu8988 cell line. As compared with the control and the LV-NC cell variants, the corresponding B7-H3 knockdown cell variant, LV-shB7-H3, expressed a low level of B7-H3 mRNA and protein (Fig. 3A and B). Following isolation by sorting FCM, stable Patu8988 cell lines in which B7-H3 had been knocked down (LV-shB7-H3) and non-targeted control mock lentivirus infected Patu8988 cells (LV-NC) were established (Fig. 3C).

**Figure 4:**
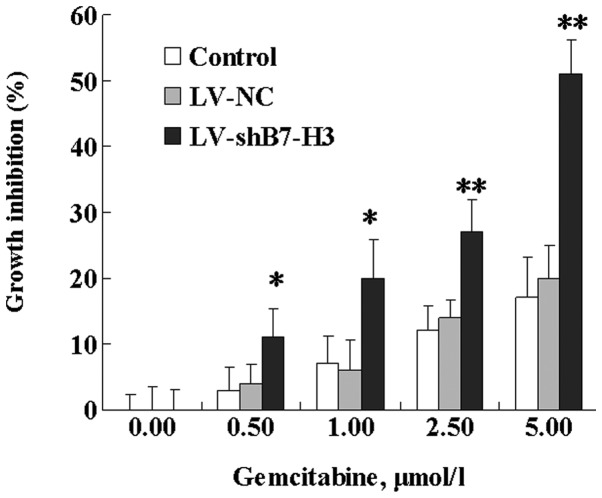
B7-H3 silencing increased sensitivity to gemcitabine in pancreatic carcinoma cells. Data are presented as the percentage of cell growth inhibition measured in gemcitabine-treated cells compared with untreated cells. The P-value indicates the significance of the difference between the Patu8988 cell lines in which B7-H3 had been knocked down (the LV-shB7-H3 group) and the control cells. Bars, mean of three independent experiments performed in triplicate (^**^P<0.05, ^***^P<0.01, vs. the control group). LV-NC, non-targeted control mock lentivirus-infected Patu8988 cells.

Following treatment with various concentrations of gemcitabine for 72 h, a dose-dependent inhibition of cell growth was observed in Patu8988 cells (Fig. 4). In Patu8988 cell variants, the inhibition of cell growth was 51% following exposure to 5.00 *μ*mol/l gemcitabine in the LV-shB7-H3 cells, compared with 17 and 20% in the control and LV-NC cells, respectively. Statistical analysis revealed that the difference in growth inhibition between LV-shB7-H3 and LV-NC cells was significant. These results indicate that B7-H3 is involved in tumor cell resistance to gemcitabine. No marked differences were observed between the LV-NC and control cells with respect to gemcitabine responsiveness.

### B7-H3 plays a critical role in cancer cell resistance to gemcitabine-induced apoptosis

**Figure 5:**
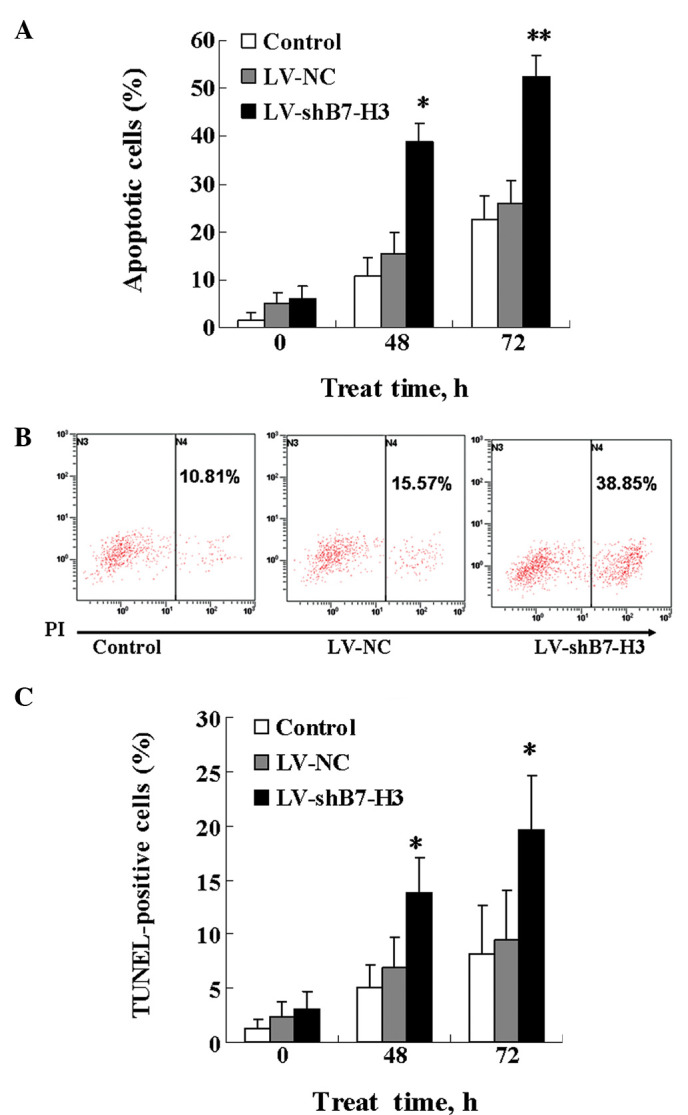
B7-H3 silencing sensitizes pancreatic carcinoma cells to gemcitabine-induced apoptosis. (A) The percentage of Annexin V-stained cells increased in the Patu8988 cell lines in which B7-H3 had been knocked down (the LV-shB7-H3 group). Apoptosis was examined by Annexin V staining and flow cytometry. (B) Flow cytometry diagram at 48 h detected by Annexin V assay. (C) The percentage of TUNEL-positive cells increased in the LV-shB7-H3 cells. Apoptosis was examined by a TUNEL assay and flow cytometry. The P-value indicates the significance of the difference between the LV-shB7-H3 cells and control cells (^*^P<0.05; ^**^P<0.01). LV-NC, non-targeted control mock lentivirus-infected Patu8988 cells; TUNEL, terminal deoxynucleotidyltransferase-mediated dUTP-biotin nick-end labeling.

Gemcitabine is known to exert its cytotoxic effect through induction of apoptosis. Therefore, we investigated whether the increased gemcitabine cytotoxicity observed in cells in which B7-H3 has been knocked down was correlated with effects on apoptosis. The extent of apoptosis in Patu8988 cells was investigated by measuring the percentage of Annexin V (PI)-stained cells, a marker for early stage apoptosis, and by measuring the percentage of TUNEL-positive cells, which reflects late-stage apoptosis. In the Annexin V assay, the response to 5.00 *μ*mol/l gemcitabine was time-dependent, with an increase in the percentage of Annexin V-positive cells detected at 48 and 72 h. The LV-shB7-H3 cells were more sensitive to gemcitabine-induced apoptosis than the control and LV-NC cells (Fig. 5A and B). Similar results were observed for the TUNEL assay. As demonstrated in Fig. 5C, the LV-shB7-H3 cells were significantly more susceptible to gemcitabine-induced apoptosis than the control and LV-NC cells; the percentage of TUNEL-positive cells was 13.76% vs. 5.07% (control) and 6.84% (LV-NC) at 48 h, and 19.64% vs. 8.21% (control) and 9.43% (LV-NC) at 72 h. Overall, these results demonstrate that silencing of B7-H3 expression by shB7-H3 causes the cells to become more prone to gemcitabine-induced apoptosis.

### B7-H3 regulates the activation of the anti-apoptotic molecule survivin

**Figure 6:**
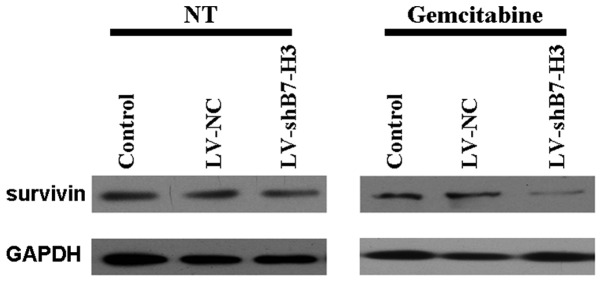
B7-H3 at least partially regulates the expression of survivin in pancreatic carcinoma cells. Cells were treated with 5.00 *μ*mol/l gemcitabine for 72 h or were left untreated. Whole cell lysates were probed with indicated antibodies with GAPDH as an internal control. B7-H3 silencing was observed to downregulate survivin expression in Patu8988 cells, particularly those treated with gemcitabine.

As chemosensitization accompanied with an increase in apoptosis was observed in gemcitabine-treated cells in which BH-73 had been knocked down, we subsequently studied whether the effects of B7-H3 may be associated with molecules known to be involved in the apoptotic response. As demonstrated in Fig. 6, the silencing of B7-H3 induced a marked reduction in the level of survivin, an anti-apoptotic factor, both in untreated and gemcitabine-treated cells. This indicates that the effect of silencing B7-H3 on increasing gemcitabine cytotoxicity was through decreased expression of the anti-apoptotic protein, survivin. This may be the result of B7-H3 regulating a certain signaling pathway.

### Silencing of B7-H3 enhances cancer cell sensitivity to gemcitabine in a xenograft mouse model

**Figure 7:**
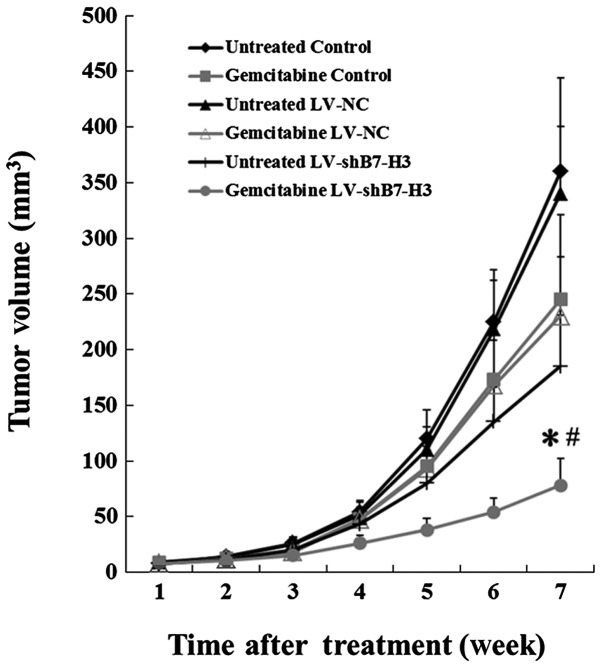
Inhibition of Patu8988 xenotransplantation tumor growth *in vivo*. Growth curves of pancreatic carcinoma xenografts in nude mice were as shown. Each group consisted of 6 animals and the data are presented as mean ± standard deviation. The tumor volume of the gemcitabine-treated Patu8988 cell lines in which B7-H3 had been knocked down (the LV-shB7-H3 group) was significantly lower (^*^P<0.01, gemcitabine LV-shB7-H3 vs. gemcitabine control; ^#^P<0.05, gemcitabine LV-shB7-H3 vs. untreated LV-shB7-H3). LV-NC, non-targeted control mock lentivirus-infected Patu8988 cells.

The *in vitro* experiments with the Patu8988 cells demonstrated that the cytotoxic effect of gemcitabine was enhanced in cells with silenced B7-H3. Hence, we examined whether this effect also occurred *in vivo*. LV-shB7-H3, LV-NC and control cells were injected subcutaneously into nude mice, and the animals were treated with gemcitabine when the tumors had reached a mean diameter of 5–6 mm. As demonstrated in Fig. 7, the growth rate was reduced by knocking down B7-H3 alone. Additionally, although gemcitabine had an effect on the growth of control group tumors, it demonstrated a strong antitumor effect in the mice carrying LV-shB7-H3 xenografts. All 36 mice developed detectable tumors at the initiation of this experiment. Inhibition of growth was observed in the gemcitabine-treated LV-shB7-H3 group for seven weeks; the average tumor volume at seven weeks was 78±24 mm^3^, which was significantly lower than that of the gemcitabine-treated LV-NC and gemcitabine-treated control groups (230±53 and 245±61 mm^3^, respectively; P<0.01). No significant differences were observed between the gemcitabine-treated LV-NC and control groups. In addition, the average tumor volume of the gemcitabine-treated LV-shB7-H3 group (78±24 mm^3^) was significantly lower than that of the untreated LV-shB7-H3 group (185±46 mm^3^; P<0.05; Fig. 7).

**Figure 8:**
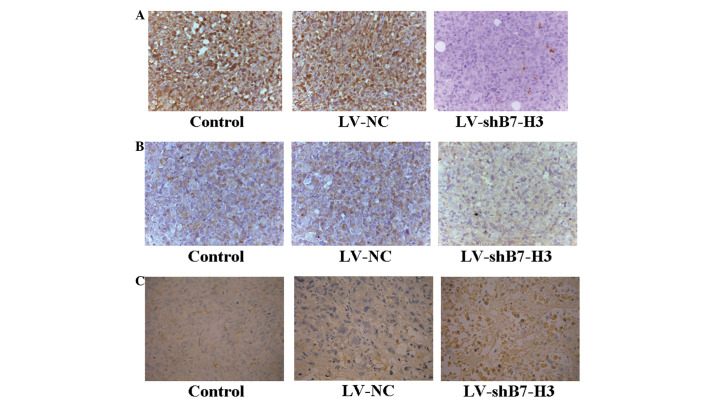
Immunohistochemical staining of a xenotransplantation tumor. (A) Expression of B7-H3. Xenografts from the gemcitabine-treated non-targeted control mock lentivirus-infected Patu8988 cells (the LV-NC group) and control group demonstrate distinct membranous and cytoplasmic immunoreactivity for B7-H3, whereas the xenograft from the gemcitabine-treated Patu8988 cell lines in which B7-H3 had been knocked down (the LV-shB7-H3 group) demonstrates weak B7-H3 expression. (B) Expression of survivin. Compared with the other two groups, the expression of survivin in the tumor of the LV-shB7-H3 group was markedly downregulated. (C) Apoptosis assay of the xenotransplantation tumor by terminal deoxynucleotidyltransferase-mediated dUTP-biotin nick-end labeling (TUNEL) staining. The number of positive apoptotic tumor cells with brown nuclei in the LV-shB7-H3 group was markedly increased compared with that of the control group and the LV-NC group. Magnification, ×400.

Knockdown in the shB7-H3 xenografts was confirmed by immunochemical staining, and while the level of B7-H3 expression remained low in the LV-shB7-H3 tumors, the LV-NC and control tumors demonstrated strong staining (Fig. 8A). In order to demonstrate the mechanism of the anti-apoptotic effect by targeting B7-H3 gene RNAi, the expression of survivin was analyzed by immunohistochemistry in nude mice transplanted tumors. The LV-shB7-H3 group (Fig. 8B) was demonstrated to have downregulated survivin expression compared with the LV-NC group. No differences between the LV-NC and control groups were observed. The TUNEL staining revealed that an increased number of apoptotic cells was evident in LV-shB7-H3 tumors treated with gemcitabine. When compared with the LV-NC group or the control group, the number of apoptotic cells in the LV-shB7-H3 group was significantly higher than that in the former two groups (Fig. 8C). This indicated that with gemcitabine treatment, inhibition of B7-H3 expression caused apoptotic cell death in pancreatic carcinoma cells *in vivo*. These *in vivo* results strongly support the effects observed *in vitro*; B7-H3 plays a critical role in responses to gemcitabine in pancreatic carcinoma cells.

## Discussion

In the present study, we examined the role of B7-H3 in gemcitabine resistance in the human pancreatic carcinoma cell line, Patu8988. Lentivirus-mediated shRNA targeting B7-H3 induced knockdown of the B7-H3 protein in the cells and resulted in increased sensitivity to the chemotherapeutic agent gemcitabine by promoting apoptosis. Furthermore, in order to demonstrate the mechanisms underlying the observed effects, we obtained the evidence for B7-H3 regulation of the key anti-apoptotic gene, survivin.

B7-H3, a member of the B7-family of molecules, is important in adaptive immune responses, and has been demonstrated to either promote or inhibit T-cell responses in various experimental systems. B7-H3 has been observed to be expressed in certain human cancer types and to be correlated with a poor outcome of cancer patients. Numerous studies have supported a role of B7-H3 in cancer progression. It was demonstrated that B7-H3 was highly expressed in human non-small cell lung cancer, and was significantly correlated with an increased risk of lymph node metastases (14). Roth *et al*(17) evaluated B7-H3 immunoreactivity in >300 patients who suffered from prostate cancer and underwent radical prostatectomy, and indicated that increased levels of B7-H3 intensity correlated with worsened clinicopathological features as well as a poorer postoperative prognosis. This result was further confirmed by an expanded sample in a tissue microarray (13). B7-H3 immunostaining represents an additional tool for the differential diagnosis of small round cell tumors and may be useful in identifying neuroblastoma patients at risk of relapse, who may take advantage of more careful follow-up (18). A previous study demonstrated that B7-H3 expression in clear cell renal cell carcinoma was present in both the tumor cells and the tumor vasculature, and represented prognostic implications (11). Thus, based on studies of previous literature, it was concluded that B7-H3 expression may play physiological and pathological roles in the oncogenesis and development of pancreatic carcinoma.

However, the exact role of B7-H3 in cancer progression remains elusive. Notably, in the present study we demonstrated that downregulation of B7-H3 reduced survivin expression. This may explain why the B7-H3 knockdown cells became more prone to gemcitabine-induced apoptosis. Survivin is a member of the family of inhibitors of apoptosis proteins (IAPs) (19–22), and is preferentially and highly expressed in cancer cells, including those of pancreatic cancer, while it is expressed at low levels in the majority of normal non-dividing adult tissues (23). The integral role of survivin in cancer cell division and survival causes it to be an attractive therapeutic target for the inhibition of cancer cell growth (19,20). It has been suggested that survivin inhibits cell death induced via the extrinsic and intrinsic apoptotic pathways, and that it confers resistance to apoptosis by directly suppressing caspase activity (24). Overexpression of survivin is correlated with resistance to gemcitabine-induced apoptosis in cancer cells. In a previous study by Yoon *et al*(25), it was demonstrated that the survivin suppressant YM155 increased human pancreatic cancer cell chemosensitivity to gemcitabine. Concomitant treatment with YM155 enhanced the chemosensitivity to gemcitabine, which was accompanied by a decrease in the expression of survivin. Knockdown of endogenous survivin via RNA interference also enhanced the sensitivity to gemcitabine. Moreover, YM155 potentiated the antitumor effect of gemcitabine in xenograft tumors of MiaPaCa-2. In a study by Hung *et al*(26), knockdown of survivin expression in a hepatocellular carcinoma cell line via short interfering RNA, increased the apoptotic cell population in such cells that had been treated with gemcitabine in comparison with scrambled control cells. Survivin knockdown resulted in a reduction of glucose-regulated protein 78 (GRP78), which may be responsible for the observed increased cell sensitivity to gemcitabine.

Notably, the effects on gemcitabine sensitivity *in vitro* were confirmed in our animal model. The growth rate of established B7-H3 knockdown xenografts was slower than that of LV-NC and the control tumors; however, the growth of these tumors was significantly inhibited by gemcitabine treatment, whereas that of LV-NC tumors was only marginally affected. Immunohistochemical analysis of the xenograft tissue confirmed that the tumors originating from shB7-H3 cells retained low expression levels of the protein, whereas the LV-NC and control tumors demonstrated strong B7-H3 staining. The expression of survivin was also downregulated in the B7-H3 knockdown xenografts. The TUNEL assay data indicated that silencing of B7-H3 induces, in parallel, reduced proliferation, and it enhances the apoptosis induced by gemcitabine.

In summary, our study investigating the role of B7-H3 in drug resistance has demonstrated that the protein confers resistance to gemcitabine *in vitro* and *in vivo* by reducing the sensitivity of pancreatic carcinoma cells to apoptosis, which is mediated via survivin expression. Furthermore, in contrast to previous studies focusing on the immunoregulatory effects of B7-H3, which is involved in the suppression of tumor immune surveillance, our data demonstrated that B7-H3 is important in determining the resistance to gemcitabine via nonimmunomechanisms, increasing gemcitabine sensitivity by promoting apoptosis. These findings provide novel insights into the role of B7-H3 in cancer and may have important implications in the development of targeted therapeutics for overcoming gemcitabine resistance. However, whether B7-H3 regulates survivin expression directly or via certain important intracellular pathways, requires further investigation.

B7-H3 is more highly expressed in pancreatic carcinoma than in normal pancreatic tissue. B7-H3 silencing in pancreatic carcinoma increases tumor cell sensitivity to gemcitabine by promoting apoptosis. Furthermore, the mechanisms underlying these effects may include that silencing B7-H3 downregulates the anti-apoptotic molecule, survivin.
